# Primary Care Provider Density and Elective Total Joint Replacement Outcomes.

**DOI:** 10.1016/j.artd.2021.05.010

**Published:** 2021-07-09

**Authors:** Bella Mehta, Collin Brantner, Nicholas Williams, Jackie Szymonifka, Iris Navarro-Millan, Lisa A. Mandl, Anne R. Bass, Linda A. Russell, Michael L. Parks, Mark P. Figgie, Joseph T. Nguyen, Said Ibrahim, Susan M. Goodman

**Affiliations:** a Department of Medicine, Hospital for Special Surgery, New York, NY, USA; b Department of Medicine, Weill Cornell Medicine, New York, NY, USA; c Department of Biostatistics and Epidemiology, Weill Cornell Medicine, New York, NY, USA; d Department of Orthopedics, Hospital for Special Surgery, New York, NY, USA; e Biostatistics Core, Hospital for Special Surgery, New York, NY, USA; f Department of Population Health Sciences, Weill Cornell Medicine, New York, NY, USA

**Keywords:** Total hip arthroplasty, Total knee arthroplasty, Primary care physicians, Provider density, WOMAC

## Abstract

**Background:**

Primary care physicians (PCPs) are often gatekeepers to specialist care. This study assessed the relationship between PCP density and total knee (TKA) and total hip arthroplasty (THA) outcomes.

**Methods:**

We obtained patient-level data from an institutional registry on patients undergoing elective primary TKA and THA for osteoarthritis, including Western Ontario and McMaster Universities Osteoarthritis Index (WOMAC) pain and function scores at baseline and 2 years. Using geocoding, we identified the number of PCPs in the patient’s census tract (communities). We used Augmented Inverse Probability Weighting and Cross-validated Targeted Minimum Loss-Based Estimation to compare provider density and outcomes adjusting for potential confounders.

**Results:**

Our sample included 3606 TKA and 4295 THA cases. The median number of PCPs in each community was similar for both procedures: TKA 2 (interquartile range 1, 6) and for THA 2 (interquartile range 1, 7). Baseline and 2-year follow-up WOMAC pain, function, and stiffness scores were not statistically significantly different comparing communities with more than median number of PCPs to those with less than median number of PCPs. In sensitivity analyses, adding 1 PCP to a community with zero PCPs would not have statistically significantly improved baseline or 2-year follow-up WOMAC pain, function, and stiffness scores.

**Conclusions:**

In this sample of patients who underwent elective TKA or THA for osteoarthritis, we found no statistically significant association between PCP density and pain, function, or stiffness outcomes at baseline or 2 years. Further studies should examine what other provider factors affect access and outcomes in THA and TKA.

## Introduction

Osteoarthritis (OA) is the most prevalent joint disease and a leading source of chronic pain and disability in the United States [1,2]. Knee and hip OA accounts for more than 80% of the disease’s total burden and affects at least 19% of American adults aged 45 years and older [3]. To date, the only definitive therapy to reduce symptoms and improve quality of life for adults with advanced hip or knee OA is joint replacement [4,5]. Although utilization of total hip (THA) or total knee arthroplasty (TKA) has increased and outcomes have improved over the past decade, these benefits are not shared equally throughout the population, and the disparity is largely mediated by social determinants of health [6]. Race and socioeconomic status are well-studied social determinants of health in arthroplasty utilization and outcomes. Race, sex, and low socioeconomic status identify groups with worse outcomes after hip and knee replacements [[7], [8], [9], [10]]. However, the nature of these relationships is complicated and may not be based on inherent or implicit biases. Of these alternative explanations, decreased access to care, appropriate orthopedic referral, timing of referral, and fewer local resources may mediate poor outcomes. Heterogeneity of local resources has been linked to timeliness of care and contributes to geographic variation in clinical outcomes. Higher proportions of providers in a geographical location (provider density) have been associated with multiple health care outcomes, including better surgical outcomes in hand and wrist surgery [11] as well as procedures for surgical emergencies such as pediatric appendicitis [12]. Specifically, primary care physician (PCP) density has been associated with improved outcomes for melanoma [13] and lung cancer [14] likely due to early recognition and appropriate referral. The availability of PCPs may be one of the contributors to outcome variability across socioeconomic gradients defined by census tract (CT) variables. Higher numbers of PCPs locally may optimize timely access to care, in turn resulting in better outcomes. Provider density may explain the variance in outcomes between communities with fewer resources. Improving access to care would mitigate differences in health care outcomes. As TKA and THA utilization are projected to significantly increase [15,16] and are among the 5 top procedures in terms of Medicare expenditures, [17] identifying modifiable risk factors for poor THA or TKA outcomes is important on a systemic level.

The American Community Survey collects data on multiple factors, including provider density, within CT. CTs are small geographical areas designed to be homogeneous and can be used to analyze community-level data to better understand health outcomes. Individual level data can be linked using geospatial localization to specific CT, allowing the study of associations of both individual- and community-level data with important health outcomes [18,19]. It is not known whether PCP density is associated with outcomes in TKA or THA.

The primary objective of our analysis is to examine the relationship of PCP density with TKA and THA outcomes. We hypothesized that patients from communities with higher proportion of PCPs would have better outcomes after primary elective hip and knee replacements.

## Material and methods

### Study design/ sample

We conducted a retrospective study using a longitudinal registry data from a high-volume orthopedic hospital whose catchment area includes the tristate area of New York, New Jersey, and Connecticut. The registry enrolled patients aged 18 years or older undergoing TKA/THA between May 1, 2007, and July 1, 2011. We included all patients who completed questionnaires after undergoing elective primary THA/TKA, specifically patient-reported outcomes, both at baseline (before TKA or THA) and 2 years after TKA or THA. We excluded registry patients who lived outside the tristate area to focus on the main catchment area for the hospital. We also excluded patients who underwent bilateral and revision procedures.

### Study covariates/ outcome

Baseline covariates collected in the registry included age at the time of surgery, sex, body mass index (BMI), race (black, white, Asian, other), ethnicity (Hispanic, non-Hispanic), and living status (alone or with others). Baseline and 2-year Knee injury and Osteoarthritis Outcome Score, from which Western Ontario and McMaster Universities Osteoarthritis Index (WOMAC) pain, function, and stiffness scores are derived for this analysis, were also collected. Patient addresses were collected from hospital records and used to permit geocoding.

Primary exposure was number of PCPs/CT (community). The primary outcomes of the study were baseline and 2-year WOMAC pain, function, and stiffness scores. The WOMAC is a validated lower extremity–specific scale and commonly used as a patient-reported instrument after TKA or THA. WOMAC results are scored on a 0- to 100-point scale, with higher scores indicating better outcomes. Lower extremity pain, function, and stiffness are assessed using 3 subscales; a higher score indicates better status, and a score less than 40 indicates significant pain, poor function, or stiffness [20].

### Census tracts/ geocoding

Geocoding is a method of converting addresses to geographic coordinates which can be used to place markers or positions on a map. From this, we can perform spatial analysis using geographic information systems and Enterprise Location Intelligence systems. The American Community Survey/Census Bureau releases multiple data sets containing area-based measures (ABMs) every 10 years. ABMs are data collected by the Census Bureau and broken down into smaller subsections to characterize the community. We presented the data using CTs (average 4000 residents). CTs are designed to be homogenous and are not affected by methodological problems plaguing socioeconomic research using ZIP code-level data [21].

Each patient in the registry with a geocodable address was assigned to a CT using ArcGIS [22]. ABMs were then extracted for each CT using data released by the Census Bureau. PCP density was calculated using the number of PCPs within each tract listed in the Census Bureau data. The tristate area has a total of 7762 CTs. Of the included CTs, 551 in the knee cohort and 569 in the hip cohort did not have any PCPs.

### Statistical analysis

Hip and knee cohorts were described using counts and percentages, and medians and interquartile ranges for discrete and continuous variables, respectively. Census-tract PCP density was dichotomized at the median for the respective patient cohort. Augmented inverse probability weighting (A-IPW) was then used to assess the effect of dichotomized census-tract PCP density on patient baseline and 2-year postoperation WOMAC function, stiffness, and pain for both cohorts [23].

The following patient-level covariates were included for adjustment: BMI, age at surgery, race, ethnicity, sex, number of comorbidities, if the patient lives alone, and insurance type; patient-level covariates were aggregated at the census-tract level. The following census-tract covariates were included for adjustment: percent less than high-school education, percent minimum high-school education, percent minimum college-degree, percent foreign born, percent speak only English, percent speak poor English, and percent living below the poverty level. Baseline WOMAC scores were not included as covariates when assessing 2-year postoperation WOMAC because they are a mediator on the exposure-outcome pathway. Missing covariates were imputed at the median.

Cross-validated Targeted Minimum Loss-Based Estimation (CV-TMLE) was then used to estimate the effect of a modified treatment policy where communities with zero PCPs per 1000 population were increased to one PCP per 1000 population on patient baseline and 2-year postoperation WOMAC function, stiffness, and pain for both cohorts as a sensitivity analysis. For this model, the following patient-level covariates were included for adjustment: BMI, age at surgery, race, ethnicity, sex, number of comorbidities, if the patient lives alone, and insurance type; patient-level covariates were aggregated at the census-tract level. The following census-tract covariates were included for adjustment: percent less than high-school education, percent minimum high-school education, percent minimum college-degree, percent foreign born, percent speak only English, percent speak poor English, and percent living below the poverty level. Baseline WOMAC scores were not included as covariates when assessing 2-year postoperation WOMAC because they are a mediator on the exposure-outcome pathway. Missing covariates were imputed at the median/mode, and right censoring was accounted for.

CV-TMLE and A-IPW were fit using a cross-validated selection algorithm that fits the best-weighted combination of different models. The following candidate models were included in estimation of either CV-TMLE or A-IPW: generalized linear models, least absolute shrinkage and selection operator, multivariate adaptive regression splines, generalized additive models, random forest, and Extreme Gradient Boosting. Readers interested in learning more about modified treatment policies and ensemble machine learning are referred to the articles by Diaz et al. and Naimi and Balzer, respectively [24,25]. All effect estimates are presented with 95% confidence intervals. All data were analyzed in R statistical software version 3.6.0 (R Foundation for Statistical Computing, Vienna, Austria).

## Results

### Sample characteristics

**Figure 1:**
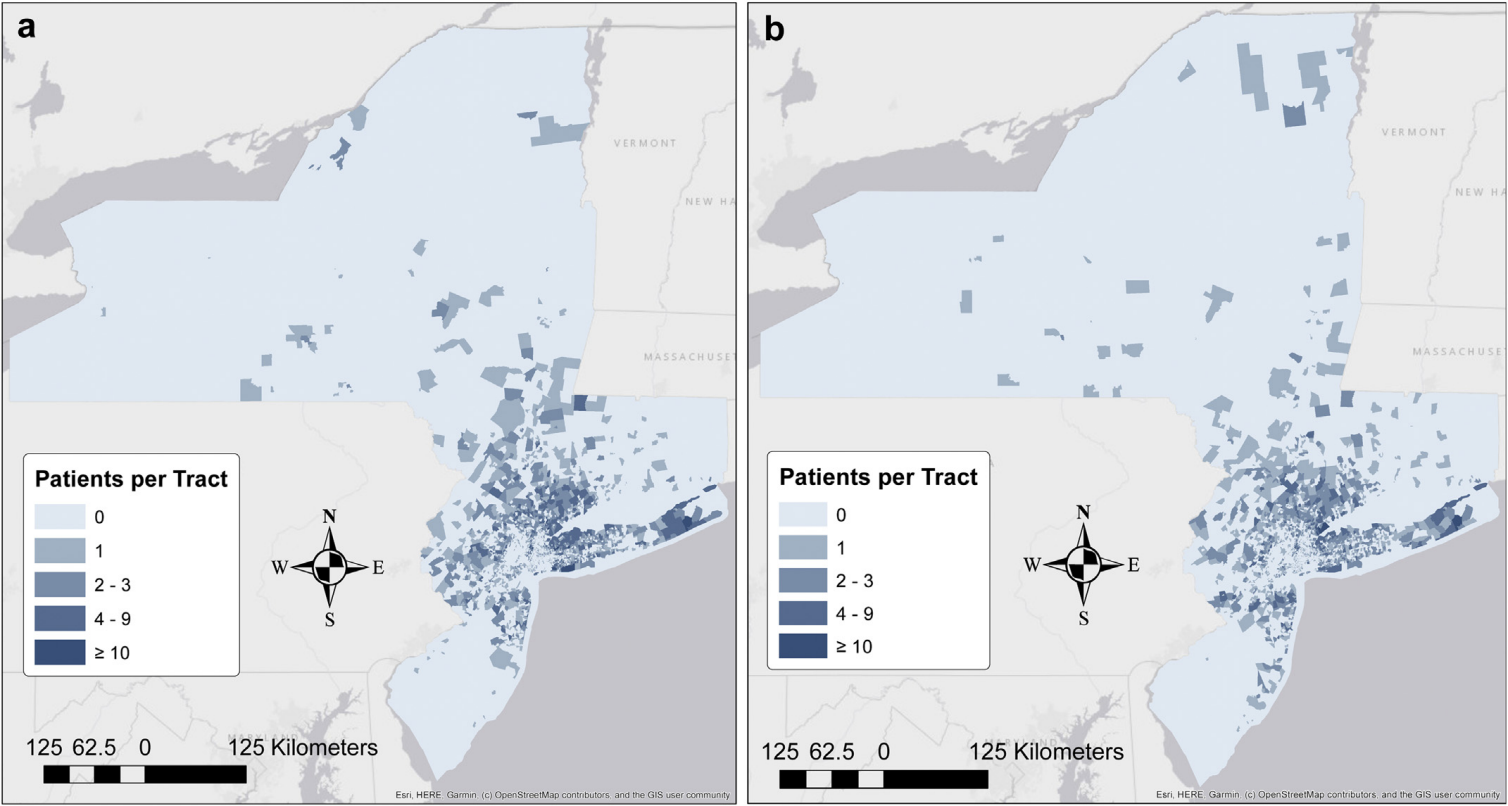
(a) Number of total hip arthroplasty patients per census tract. (b) Number of total knee arthroplasty patients per census tract.

A total of 3606 TKA and 4295 THA patients belonging to 1894 and 1961 unique CTs, respectively, were included in the analysis. (Table 1) (Fig. 1a and b). Of the total TKA patients, 2225 (61.7%) were female, 2164 (60.0%) had an undergraduate college degree or higher, and 3199 (88.7%) were white. The median number of PCPs per CT was 2 (1, 6). The median proportion of PCPs per population of 1000 was 0.4 (0.1, 1.2). Of the total THA patients, 2389 (55.6%) were female, 2880 (69.6%) had an undergraduate college degree or higher, and 3983 (93.13%) were white. The median number of PCPs per CT was 2 (1, 7). The median proportion of PCPs per population of 1000 was 0.4 (0.1, 1.6) (Table 2).

**Table 1:** Patient and census tract demographics and characteristics.

Characteristic	THA			TKA		
	Overall	Below median	Above median	Overall	Below median	Above median
Patient level						
Age at surgery	66 (58, 73)	65 (57, 73)	66 (58, 74)	68 (61, 75)	68 (61, 75)	69 (61, 75)
Female	2389 (56%)	1044 (55%)	1345 (57%)	2225 (62%)	1025 (62%)	1200 (61%)
White	3983 (93%)	1812 (92%)	2171 (94%)	3199 (89%)	1460 (88%)	1739 (91%)
Hispanic	108 (2.5%)	50 (2.5%)	58 (2.5%)	127 (3.5%)	64 (3.8%)	63 (3.3%)
Live alone	947 (22%)	393 (20%)	554 (24%)	823 (23%)	368 (22%)	455 (24%)
Number of comorbidities	6 (4, 8)	6 (4, 8)	6 (4, 8)	7 (5, 9)	7 (5, 9)	7 (5, 9)
Medicare	2391 (56%)	1061 (54%)	1330 (57%)	2352 (65%)	1067 (64%)	1285 (67%)
Medicaid	66 (1.5%)	35 (1.8%)	31 (1.3%)	76 (2.1%)	52 (3.1%)	24 (1.2%)
Commercial	4187 (97%)	1919 (97%)	2268 (98%)	3507 (97%)	1627 (97%)	1880 (97%)
Self-pay	1985 (46%)	948 (48%)	1037 (45%)	1322 (37%)	613 937%)	709 (37%)
Census level						
<HS% (IQR)	2.8 (1.3, 5.7)	3.0 (1.3, 6.1)	2.7 (1.3, 5.5)	3.1 (1.3, 6.2)	3.2 (1.3, 6.8)	2.9 (1.3, 5.8)
% HS	93 (87, 96)	92 (86, 96)	93 (88, 96)	92 (86, 96)	92 (86, 95)	93 (87, 96)
% Bachelors	43 (30, 60)	39 (27, 56)	47 (33, 63)	41 (29, 58)	38 (26, 53)	46 (32, 63)
% Foreign born	17 (10, 29)	16 (8, 30)	17 (10, 28)	18 (10, 29)	16 (8, 30)	18 (11, 29)
% Only speak English	78 (62, 87)	80 (60, 89)	78 (63, 85)	77 (62, 87)	80 (61, 88)	76 (63, 85)
% Speak poor English	6 (3, 14)	6 (3, 15)	7 (4, 13)	7 (3, 15)	6 (3, 16)	7 (4, 14)
% Speak other language	22 (13, 38)	20 (11, 40)	22 (15, 37)	23 (13, 38)	20 (12, 39)	24 (15, 37)
% Below poverty line	5 (2, 10)	5 (3, 10)	5 (2, 9)	5 (2, 10)	5 (3, 11)	5 (2, 10)

IQR, interquartile range; HS, high-school educated.

Statistics presented: median (IQR); n (%).

**Table 2:** Observed preoperation and follow-up WOMAC by patient cohort and census tract primary care provider density.

Cohort	Census tract PCPs (n/1000)	Preoperation			2 years		
		Function	Pain	Stiffness	Function	Pain	Stiffness
Hip	Below median [0, 0.405], N = 1977, K = 981	48 (37, 60)	50 (40, 65)	38 (25, 50)	96 (87, 100)	100 (90, 100)	100 (75, 100)
	Above median [0.405, 82.7], N = 2318, K = 980	50 (38, 63)	55 (45, 65)	50 (25, 63)	97 (88, 100)	100 (90, 100)	100 (75, 100)
Knee	Below median [0, 0.385], N = 1676, K = 947	51 (41, 65)	55 (40, 65)	50 (38, 62)	91 (75, 97)	95 (80, 100)	75 (62, 100)
	Above median [0.385, 125], N = 1930, K = 947	54 (44, 68)	55 (45, 69)	50 (38, 62)	91 (78, 98)	95 (80, 100)	75 (62, 100)

Statistics presented: Median (IQR).

K = # of Census Tracts.

### Results from multivariable models

When estimating the difference in baseline WOMAC pain, function, and stiffness scores between the entire patient population living in a CT above the median PCP density vs below the median PCP density, no statistically significantly different effect was found in either TKA (baseline WOMAC pain, function, and stiffness average treatment effect *P* = .78, .52, and 0.95, respectively) nor THA patients (baseline WOMAC pain, function, and stiffness average treatment effect *P* = .73, .58, and 0.38, respectively). No difference in PCP provider density effect was detected in 2-year follow-up scores for TKA (2-year WOMAC pain, function, and stiffness average treatment effect *P* = .93, .93, and 0.78, respectively) nor THA patients (2-year WOMAC pain, function, and stiffness average treatment effect *P* = .75, .87, and 0.96, respectively).

### Results from sensitivity analysis

In sensitivity analysis using CV-TMLE, when focusing on CTs with zero PCPs (hip cohort n = 569; knee cohort n = 551), adding one provider resulted in no significant effect on WOMAC function, pain, or stiffness at the time of surgery in the hip cohort (*P* = .47, .9, and 0.18, respectively) or in the knee cohort (*P* = .93, .21, and 0.2, respectively) (Table 3). Similarly, no significant treatment effect was observed in 2-year WOMAC scores for hip (*P* = .85, .78, and 0.72, respectively) or knee (*P* = .98, .66, and 0.98, respectively) cohorts (Table 4).

**Table 3:** Results of TMLE for effect of census tract provider density increase on patient preoperation WOMAC among both patient cohorts.

Hip cohort	Observed PCP density		Treatment policy		Treatment policy effect		
	Estimate	95% CI	Estimate	95% CI	Estimate	95% CI	*P*
Function	49.7	(44.4, 55.0)	49.8	(44.6, 55.1)	0.095	(−0.16, 0.35)	.47
Pain	53.4	(47.9, 58.9)	53.4	(47.9, 58.9)	−0.017	(−0.27, 0.24)	.9
Stiffness	45.2	(40.4, 50.0)	45.4	(40.6, 50.1)	0.2	(−0.09, 0.49)	.18
Knee cohort							
Function	53.97	(49.47, 58.48)	53.96	(49.49, 58.43)	−0.01	(−0.27, 0.25)	.93
Pain	54.79	(50.27, 59.30)	54.62	(50.15, 59.08)	−0.17	(−0.43, 0.09)	.21
Stiffness	46.58	(42.55, 50.60)	46.38	(42.40, 50.35)	−0.2	(−0.50, 0.11)	.2

CI, confidence interval.

**Table 4:** Results of TMLE for effect of census tract provider density increase on 2-year patient follow-up WOMAC among both patient cohorts.

Hip cohort	Observed PCP density		Treatment policy		Treatment policy effect		
	Estimate	95% CI	Estimate	95% CI	Estimate	95% CI	*P*
Function	90.8	(81.9, 99.6)	90.7	(81.9, 99.6)	−0.08	(−1.00, 0.85)	.87
Pain	93.5	(84.4, 100)	93.5	(84.3, 100)	−0.14	(−0.96, 0.69)	.75
Stiffness	88.4	(79.7, 97.1)	88.4	(79.7, 97.1)	−0.03	(−1.23, 1.17)	.96
Knee cohort							
Function	84.9	(78.48, 91.32)	84.9	(78.50, 91.30)	0	(−0.25, 0.26)	.98
Pain	87.4	(80.81, 94.02)	87.47	(80.89, 94.05)	0.05	(−0.19, 0.30)	.66
Stiffness	77.6	(71.64, 83.58)	77.61	(71.67, 83.55)	0	(−0.31, 0.32)	.98

CI, confidence interval.

## Discussion

In this analysis of patients undergoing elective total joint replacement, we found no statistically significant association between PCP provider density and patient-reported pain, stiffness, or function, at the time of surgical procedure or 2 years later. Furthermore, having less than median number of PCPs in a community did not increase the probability of worse baseline or 2-year postoperative outcomes. Finally, theoretically having one additional PCP in a community that previously had zero would not have resulted in any improvement in baseline or 2-year WOMAC scores in patients who underwent elective hip and knee joint replacement.

To our knowledge, only 4 other studies have examined the relationship between provider density and medical and/or surgical interventions. Although no previous studies have looked at the relationship between provider density and total joint replacements, our results differ from those that have been previously reported. One study found that in areas with higher poverty rates, provider density mediated the prescription rate of antibiotics [26]. Camp et al. analyzed the National Inpatient Sample database and the Kids’ Inpatient Database and found that in the highest quartile of physician density, pediatric patients had a significantly lower likelihood of being admitted with a perforated appendix than with acute appendicitis alluding to the fact that physicians can recognize symptoms in a timely manner and refer patients to the hospital before complications arise (odds ratio = 0.88; 95% confidence interval = 0.78 – 0.99) [12]. Backhus et al. showed that higher PCP density was associated with a reduction in lung cancer mortality for white people by a factor of 4.1 per 100,000 [14]. Finally, Fleming et al. found that higher PCP density corresponded to a greater diagnosis rate of melanoma without experiencing a decrease in diagnosis of end-stage disease [13]. We hypothesized that we would see a similar trend in the relationship between PCPs and total hip or knee replacements because appropriate timing of surgery in end-stage OA may affect outcomes [27]. Specifically, delaying surgery may contribute to worse outcomes. However, we found no significant relationship between the 2. This may be because in high-volume centers, such as ours, community factors have less of an impact on health outcomes than lower volume centers, where most TKAs and THAs are performed [28,29].

There are several limitations to consider when interpreting our results. First, our data are from a single, albeit high-volume, orthopedic hospital and may not be generalizable to other hospitals or regions of the country. Second, only patients who consented to be in the study and filled questionnaires were included, which introduces the possibility of selection bias. This could underestimate the number of poorer outcomes as those with worse outcomes may be less likely to return questionnaires. Finally, we only examined one patient-centered outcome survey. While the outcome measure did collect key constructs of pain, function, and stiffness, we did not examine other relevant outcomes such as postdischarge complications, readmission to acute hospital, or discharge destinations for rehabilitation (home vs institution). While we acknowledge the limitations, a key strength in our study was that we were able to analyze a relatively large sample size of patients from a well-validated and robust TKA and THA registry with patient-reported outcomes from one of the leading orthopedic institutions in the country.

## Conclusions

In this cohort of elective total hip and knee replacement patients, we found no statistically significant association between PCP provider density and patient-reported pain, function, or stiffness at baseline or 2 years. Future studies should look at the density of specialists, including orthopedic surgeons, to determine if there are relationships between specialty care providers and total hip or knee arthroplasty outcomes.

## Conflicts of interest

The authors declare that they have no known competing financial interests or personal relationships that could have appeared to influence the work reported in this article.

## Appendix A Supplementary data

Conflict of Interest Statement for Bass

Conflict of Interest Statement for Brantner

Conflict of Interest Statement for Figgie

Conflict of Interest Statement for Goodman

Conflict of Interest Statement for Ibrahim

Conflict of Interest Statement for Mandl

Conflict of Interest Statement for Mehta

Conflict of Interest Statement for Navarro-Millan

Conflict of Interest Statement for Nguyen

Conflict of Interest Statement for Parks

Conflict of Interest Statement for Russell

Conflict of Interest Statement for Szymonifka

Conflict of Interest Statement for Williams
