# Supplementary material for: Primary Care Provider Density and Elective Total Joint Replacement Outcomes
Source: Arthroplast Today. 2021 Jul 9;10:73–8. doi: 10.1016/j.artd.2021.05.010 (PMC8430425; doi:10.1016/j.artd.2021.05.010)
Supplement: Conflict of Interest Statement for Figgie [file mmc3.pdf]

CONFLICT OF INTEREST STATEMENT

American Association of Hip and Knee Surgeons

(Adopted from the American Academy of Orthopaedic Surgeons disclosure statement)

The following form **must be filled out completely and submitted by each author (example, 6 authors, 6 forms). All items require a response. If there is no relevant disclosure for a given item, enter "*None.*"**

Primary Care Provider Density and Elective Total Joint Replacement Outcomes.

Manuscript Title

1. Royalties from a company or supplier (The following conflicts were disclosed)

IP royalties from Lima and Wishbone

2. Speakers bureau/paid presentations for a company or supplier (The following conflicts were disclosed)

None

3A. Paid employee for a company or supplier (The following conflicts were disclosed)

None

3B. Paid consultant for a company or supplier (The following conflicts were disclosed)

Paid consultant for Lima and Wishbone

3C. Unpaid consultants for a company or supplier (The following conflicts were disclosed)

None

4. Stock or stock options in a company or supplier (The following conflicts were disclosed)

Stock in HS2, Insight, Mekanika, Wishbone

5. Research support from a company or supplier as a Principal Investigator (The following conflicts were disclosed)

None

6. Other financial or material support from a company or supplier (The following conflicts were disclosed)

None

7. Royalties, financial or material support from publishers (The following conflicts were disclosed)

None

8. Medical/Orthopaedic publications editorial/governing board (The following conflicts were disclosed)

None

9. Board member/committee appointments for a society (The following conflicts were disclosed)

None

Each author must sign AND print or type his/her name, date and submit a separate form

In addition, one BLINDED Conflict of Interest form (no author names used) should be submitted per manuscript with all author disclosures.

Mark Figgie, MD	[signature]	11/25/2020
Author Name (Print or Type)	Author Signature	Date
